# Notes and News

**Published:** 1901-07-27

**Authors:** 


### NOTES AND NEWS.

The Metropolitan Hospital Sunday Fund already exceeds £51,000 as the result of this year's collection.

Sir Robert and Lady Pullar have handed over to the people of Dundee their munificent gift of a completely equipped hospital for consumption.

The Blue Book containing the first report of the Royal Commission to inquire into arsenical poisoning from the consumption of beer and other articles of food and drink has just been issued. The Commissioners state that they regard it essential to institute further inquiry before recommending the standard test which should be imposed.

The following appointments to the Royal Victorian Order have just been made:—Sir Thomas Smith, Deputy Surgeon-General H. Blanc, and W. H. Bennet, Esq., F.R.C.S., have been created Knight Commanders of the Order; C. Hood, Esq., M.D., J. H. Morgan, Esq., F.R.C.S., and C. A. Morris, Esq., F.R.C.S., have been created Commanders of the Order.

The Orient liner *Ormuz*, from Sydney, which was not allowed to put in at Marseilles or Gibraltar owing to cases of plague being reported on her, has arrived at Plymouth, and the two cases were immediately transferred to a port hospital ship, where they will remain until all doubt as to the nature of their illness is removed. The port medical officers carefully made a list of the names and destinations of the passengers who landed at Plymouth.

On Tuesday in next week the annual meeting of the British Medical Association opens at Cheltenham under the presidency of Dr. Elliston, consulting surgeon to the East Suffolk and Ipswich Hospital; the President-elect being Dr. George Bagot Ferguson, senior surgeon to the Cheltenham Hospital. The address in medicine will be delivered by Dr. Goodhart, consulting physician to Guy's Hospital, and that in surgery by Sir William Thompson, hon. surgeon to the King in Ireland.

Mr. W. Astor has given £1,000 towards securing a home for the nurses at the Friedenheim Hospital. Sir F. and Lady Wills have given £500, and the Duke of Bedford £100 towards the same object.

Last week Sir John Burdon Saunderson, Regius Professor of Medicine at Oxford, opened the New Physical Laboratory at the University of Edinburgh. This laboratory has been established by Mrs. Cox in memory of her father, Professor Hughes Bennett, and it was especially appropriate that one of Professor Hughes Bennett's most eminent pupils should open the buildings.

A lamentable accident which occurred a week or two ago, while showing how widespread is unselfish, and we might say reckless, daring among Englishmen of all degrees, demonstrates, at the same time, how futile has been all the elementary science teaching with which the land has been flooded for the last thirty years. At the Three Mills Distillery, West Ham, there is a well which had not been inspected for a long time, and Mr. G. M. Nicholson, the managing director of the distillery, accompanied a party to ascertain what depth of water it contained. The cover was removed, and a man descended by a ladder, and after ascertaining the depth of the water he suddenly fell to the bottom of the well. Mr. Nicholson at once went down the ladder to his assistance, and he, too, as soon as he got down a little way, was overcome by the foul air and fell. Then the foreman descended, and he fell also; then went down another workman to meet the same fate; and, finally, still another man descended, but was lucky enough to be got out quickly, and so just escaped the fate of the others. But is it not strange that such an accident should happen at this time of day, not among ignorant labourers, but among people of some position and intelligence? Four good men killed, one after the other, just for lack of the precaution of testing a well by sending down a lighted candle before going down themselves.

At a recent meeting of the Metropolitan Asylums Board the Hospitals Committee presented a report showing in detail the accommodation which, after conference with the London County Council and at the request of the Local Government Board, they had set aside for dealing with suspects and plague cases in London, and for providing which an expenditure of £1,500 had so far been incurred. It is to be noted that it has been arranged that no certificates, whether in respect of a case of plague or a suspect, will be accepted by the managers unless signed by one of certain experts named for the purpose by the London County Council. Of course, all this complicated interlocking of authorities is absolutely absurd, and it is to be taken for granted that, if London should have the misfortune to be seriously infected with plague, some single authority—probably the Local Government Board—will have to take the reins. The misfortune is that in dealing with such a disease everything depends upon rapid and efficient action on the first outbreak, and action is not likely to be either rapid or efficient while the responsibility is divided between so many bodies—the local authorities, the County Council, the Metropolitan Asylums Board, and the Local Government Board. Surely the time has arrived when there ought to be one central health authority for London with full and unhampered power to act.

